# Sleep Related Movement Disorders: What's New and Changing Clinical Practice

**DOI:** 10.1111/jsr.70210

**Published:** 2025-10-03

**Authors:** Ambra Stefani, Qi Tang, Stefan Clemens, Lourdes M. DelRosso, Diego Garcia‐Borreguero, Raffaele Ferri, Birgit Frauscher, Evi Holzknecht, Federica Provini, Barbara Schormair, John Winkelman, Birgit Högl

**Affiliations:** ^1^ Medical University Innsbruck Innsbruck Austria; ^2^ East Carolina University Greenville North Carolina USA; ^3^ University of California San Francisco Fresno California USA; ^4^ Sleep Research Institute Madrid Spain; ^5^ Sleep Research Centre, Department of Neurology IC Oasi Research Institute – IRCCS Troina Italy; ^6^ Duke University Durham North Carolina USA; ^7^ Department of Biomedical and NeuroMotor Sciences University of Bologna Bologna Italy; ^8^ IRCCS Istituto delle Scienze Neurologiche di Bologna Bologna Italy; ^9^ Institute of Neurogenomics Computational Health Department Helmholtz Munich, Munich Germany; ^10^ Institute of Human Genetics, TUM School of Medicine and Health Technical University Munich Munich Germany; ^11^ Departments of Psychiatry and Neurology Massachusetts General Hospital Boston Massachusetts USA

**Keywords:** large muscle group movements, restless legs syndrome, restless sleep disorder, RLS, RSDLMM

## Abstract

Restless legs syndrome (RLS) is a common sensorimotor disorder, and the most common sleep‐related movement disorder with a prevalence of up to 15% in the European and US population. This review addresses key aspects of RLS, focusing on novel data that have or will likely have an impact on clinical practice. These include novel insights into pathophysiology and motor activity during sleep, with a key focus on implications for RLS treatment. Along this line, we discuss the problem of augmentation before introducing new treatment paradigms and insights into new drug targets from genetics. Besides RLS, restless sleep disorder, neck myoclonus, fragmentary myoclonus, propriospinal myoclonus at the wake–sleep transition, and facio‐mandibular myoclonus are discussed. This review provides an overview of the most recent insights into sleep‐related movement disorders, and of how they are changing clinical practice.

## 1 Introduction

Restless legs syndrome (RLS) is a common sensorimotor disorder and the most common sleep‐related movement disorder, with a prevalence of up to 15% in the European and US populations (Broström et al. 2023). It is characterised by an urge to move the legs, usually accompanied by or felt to be caused by unpleasant sensations in the legs, appearing or worsening in the evening and/or at night and at rest, and improving with movement (at least as long as the movement persists) (Allen, Picchietti, et al. 2014). As RLS diagnosis is clinical, a key challenge is the exclusion of other conditions, which could mimic RLS symptoms (Allen, Picchietti, et al. 2014). This review addresses key aspects of RLS, focusing on novel data that have or will likely have an impact on clinical practice. These include novel insights into pathophysiology and motor activity during sleep (beyond periodic leg movements during sleep, PLMS), with a key focus on implications for RLS treatment. Along this line, we discuss the problem of augmentation before introducing new treatment paradigms and insights into new drug targets from genetics.

Besides RLS, restless sleep disorder (RSD), neck myoclonus (NM), fragmentary myoclonus (FM), propriospinal myoclonus (PSM) at the wake–sleep transition, and facio‐mandibular myoclonus (FMM) are discussed. RSD has been described recently as a new sleep disorder in children, and its relevance in the adult population is just starting to be investigated. Potential clinical implications of NM and FM, and the relevance of PSM and FMM are discussed based on recent literature.

This review provides an overview of the most recent insights into sleep‐related movement disorders, and of how they are changing clinical practice.

## 2 Restless Legs Syndrome

### 2.1 Recent Insights Into Pathophysiology

#### 2.1.1 Overview

RLS is a common neurological disorder that is clinically significant in 2%–5% of the general population (1). Yet, despite this high prevalence, its underlying pathophysiology is only partially understood. RLS is a complex neurological disorder that strongly affects sleep, with both supraspinal and spinal components, and it is associated with genetic and environmental factors contributing to its development.

RLS often exhibits a familial pattern, suggesting a genetic component. Genome‐wide association studies have identified over 160 risk loci associated with RLS (Schormair et al. 2024). However, no single gene has been found to be highly penetrant, suggesting either that multiple genes likely interact to influence RLS susceptibility or that these genes independently target overlapping cellular pathways.

RLS is also strongly associated with disruptions in iron‐related neural homeostasis (Earley et al. 2014). CNS iron plays a crucial role in various neurological functions, and its deficiency or dysregulation can have significant consequences for the development of symptoms. Ferroportin, a transmembrane protein that transports iron from the inside to the outside of the cell, has been implicated in the emergence of RLS symptoms (Earley et al. 2014; Connor et al. 2004), but the mechanistic links between iron metabolism and genetic risk factors for RLS remain an area of active research.

#### 2.1.2 Neurotransmitter Dysfunction

Clinical and preclinical research has provided evidence of—mostly subtle—dopaminergic abnormalities in individuals with RLS or in animal models of the disease (Earley et al. 2014; Connor et al. 2009). Hence, it remains unclear whether these abnormalities are a primary cause of RLS or rather secondary to other underlying pathologies. However, treatment with dopaminergics has been extremely successful in alleviating the symptoms, at least initially, suggesting a potential role of dopamine in the disorder.

Iron‐deficient animal models of RLS suggest the presence of hyperactive glutamatergic corticostriatal pathways, but it remains unclear if this upregulation is causal to RLS or a consequence of other changes. In animal models, brain iron deficiency is associated with an increased sensitivity of the corticostriatal terminals to release glutamate and, secondarily, dopamine, which seems to be dependent on the downregulation of inhibitory adenosine A1 receptors (A1Rs) localised in the glutamatergic neurons (Rodrigues et al. 2022; Yepes et al. 2017; Quiroz et al. 2016; Ferré et al. 2019). Drugs that increase extracellular adenosine (e.g., dipyridamole) or reduce glutamatergic function (e.g., anti‐epileptics) can improve RLS symptoms (Garcia‐Borreguero et al. 2018, 2021, 2017).

#### 2.1.3 Implications for Treatment

##### 2.1.3.1 Dopaminergic Medications

As mentioned above, over the short term, RLS is highly responsive to low‐dose treatment with dopaminergic medications. These medications target dopamine receptors, particularly those belonging to the D2/D3 receptor subtypes. However, long‐term treatment with these drugs commonly leads to a worsening of the symptoms termed augmentation (Clemens et al. 2025), which has led to a recommendation to refrain from using these drugs as first‐line treatments (Winkelman et al. 2025b; Garcia‐Borreguero et al. 2016). These aspects are discussed later.

##### 2.1.3.2 Iron‐Based Medications

Iron treatment, either oral or intravenous, can serve as a valuable treatment option to improve symptoms of RLS, especially in individuals with low iron levels. Intravenous iron is usually more effective than oral iron, as it bypasses the gastrointestinal system (Allen et al. 2018).

##### 2.1.3.3 Glutamatergic and Adenosinergic Medications

Gabapentinoids have proved to be effective therapeutic agents and act by means of a reduction of the presynaptic release of glutamate (Winkelman et al. 2025a). Similarly, antagonists of the glutamate–AMPA receptor, such as the anti‐epileptic perampanel (Garcia‐Borreguero et al. 2017), have also been reported to be effective in treating RLS symptoms.

### 2.2 Motor Activity During Sleep in RLS: Beyond PLMS

A hallmark of RLS is the presence of periodic leg movements during sleep (PLMS); while PLMS has been extensively studied, recent research has identified another category of motor activity during sleep known as large muscle group movements (LMMs), which may have significant implications for understanding and managing RLS.

In 2021, the International RLS Study Group published a position statement outlining criteria for scoring LMM during sleep. LMM is defined as movements lasting between 3 and 45 s in adults, characterised by an increase in electromyographic activity and/or movement artefacts in any combination of at least two recommended channels (Ferri et al. 2021). These movements are often accompanied by changes in sleep stage, arousals, awakenings, and increases in heart rate (Ibrahim et al. 2023; Mogavero, Congiu, Lanza, Ferini Strambi, et al. 2025).

Building upon this framework, a 2024 study investigated the characteristics of LMM in patients with RLS compared to healthy controls (Mogavero et al. 2024). The study assessed various types of leg movements during sleep, including short‐interval leg movements, PLMS, isolated leg movements (ISOLMS), and LMM. The findings revealed that all movement measures were significantly higher in the RLS group. Notably, a significant positive correlation was found between the LMM index and the ISOLMS index, but not with the PLMS index, in both groups. In RLS patients, a higher LMM index was associated with reduced total sleep time, lower sleep efficiency, decreased percentages of sleep stages N3 and REM, increased number of awakenings, and higher percentages of lighter sleep stages. These associations suggest that LMM contributes to sleep instability and fragmentation in RLS patients.

The identification and characterisation of LMM in RLS patients highlight the complexity of motor activities during sleep beyond the traditional focus on PLMS. While PLMS have been the primary target in the assessment and treatment of RLS‐related sleep disturbances, the presence of LMM suggests additional mechanisms contributing to sleep disruption. Importantly, the above study found no significant correlation between the LMM index or the PLMS index and RLS severity, indicating that LMM may affect sleep architecture independently of the subjective severity of RLS symptoms (Mogavero et al. 2024).

These findings underscore the need for a comprehensive assessment of motor activities during sleep in RLS patients, considering both PLMS and LMM. The recognition of LMM as a distinct phenomenon opens avenues for further research into their pathophysiology, clinical significance, and potential treatment implications. As LMM do not respond to dopaminergic drugs—commonly used for RLS—nor to clonazepam, if their clinical relevance in RLS is confirmed in future studies, alternative therapeutic strategies may be required to address this aspect of motor activity during sleep in RLS patients (Mogavero, Congiu, Lanza, Marelli, et al. 2025).

In conclusion, the exploration of LMM provides a more comprehensive understanding of motor activity during sleep in RLS, moving beyond the traditional focus on PLMS. The recent findings have been instrumental in defining and characterising LMM, highlighting their potential impact on sleep architecture.

### 2.3 The Problem of Augmentation and Implications for Treatment

Augmentation describes an iatrogenic worsening of RLS symptoms compared to the time before treatment was started. It represents a paradoxical response to medication, mainly dopaminergic drugs. RLS augmentation is characterised by the need for higher dosages of medication to achieve symptoms' control, an earlier onset of symptoms during the day, a shorter latency to symptoms at rest, spreading of the RLS symptoms to other body parts than those initially involved when RLS was diagnosed, greater intensity of symptoms, and a shorter medication effect (García‐Borreguero et al. 2007).

Augmentation was first recognised and is most common under levodopa (Garcia‐Borreguero et al. 2016; Allen and Earley 1996; Högl et al. 2010), followed by short‐acting and long‐acting dopamine agonists (Garcia‐Borreguero et al. 2016; Liu et al. 2016). The risk of augmentation increases with treatment duration (Garcia‐Borreguero et al. 2016; Liu et al. 2016; Allen, Chen, et al. 2014) and dopaminergic dose (Heim et al. 2022).

The mechanisms leading to augmentation are still not fully understood but include a postsynaptic adjustment to circadian and iatrogenic changes due to dopaminergic stimulation. In particular, there is a pre‐synaptic hyperdopaminergic state and a post‐synaptic receptor down‐regulation in people with RLS, with consequent relative dopamine deficit in the evening and at night (due to circadian changes in dopamine levels) (Earley et al. 2006). Iatrogenic dopaminergic stimulation acts on these dysregulated dopamine pathways (Earley et al. 2014), resulting in a vicious circle where increasing doses of dopaminergic medication not only does create more symptoms temporarily but also cause a persisting worsening of the underlying pathology (Allen 2015).

An increased awareness for augmentation led to changes in RLS treatment recommendations, moving away from dopaminergic substances and towards other drugs. Levodopa is not recommended as a long‐term treatment anymore (Winkelman et al. 2025b; Garcia‐Borreguero et al. 2016; Trenkwalder et al. 2024). In the latest version of the International Restless Legs Syndrome Study Group (IRLSSG) guidelines, alpha‐2‐delta ligands are recommended as initial treatment, with low‐dose prolonged‐release dopamine agonist as an alternative. If treatment with dopaminergic drugs is chosen, it should be used for the shortest possible time and at the lowest possible dosage (Garcia‐Borreguero et al. 2016). Recently published recommendations of the American Academy of Sleep Medicine (AASM) provide a conditional recommendation against levodopa and dopamine agonists in the treatment of RLS, although exceptions are listed (Winkelman et al. 2025b).

In clinical practice, RLS patients treated with dopaminergic drugs are still common and need to be monitored closely for features of augmentation (Winkelman et al. 2025b; Garcia‐Borreguero et al. 2016). Moreover, dopaminergic load should be kept as low as possible and the possibility to reduce it and/or switch to other treatments should be evaluated periodically.

### 2.4 New Treatment Paradigms

With increasing recognition of the long‐term complications associated with dopaminergic medications, the role of alternative approaches to the treatment of RLS has become more pressing. The three well‐established pharmacological alternatives to dopaminergic medications are: alpha‐2‐delta calcium ligands (gabapentinoids), iron treatments, and opioids.

Gabapentinoids have been recognised as effective treatments for RLS for nearly 30 years (Mellick and Mellick 1996). They bind to alpha‐2‐delta calcium channels (Hendrich et al. 2008) and in that way modify neuronal signalling. Although gabapentin, pregabalin, and gabapentin enacarbil each have data in support of their efficacy for RLS (Winkelman et al. 2025b), only the latter has FDA approval for that indication but is not available in other countries such as Europe. Pregabalin and gabapentin can only be prescribed off‐label for RLS treatment in many countries outside the USA. In addition to their benefit for core RLS symptoms, they have demonstrated improvements in sleep quality, reductions in wake after sleep onset, and reductions in PLMS (Winkelman et al. 2025b). These medications do differ in important ways: gabapentin has nonlinear pharmacokinetics, thus limiting the absorption of individual doses; gabapentin enacarbil and pregabalin have linear pharmacokinetics, the former by being a controlled release prodrug. All three agents are excreted by the kidneys. Their side effects are similar and include sedation, gait instability, cognitive impairment, dizziness, and weight gain.

Iron supplementation has been employed in the treatment of RLS for over 70 years (Nordlander 1953). Recent clinical trials demonstrate the efficacy of both oral iron and various formulations of intravenous iron, for RLS severity as well as for RLS‐related quality of life and sleep quality (Winkelman et al. 2025b). The best data for IV iron come from formulations of ferric carboxymaltose, iron dextran, and ferumoxytol, each in doses of 1000 mg. Oral iron (60 mg) is best absorbed on an empty stomach with vitamin C, taken every other day. Intravenous iron supplementation should be used when there is poor tolerability of per os iron or when ferritin levels are greater than 75 mcg/dL. Intravenous iron has a low rate of side effects, with minor infusion reactions seen at times, but anaphylaxis is exceedingly rare.

Low dose opioids have been recognised as an effective treatment for RLS for hundreds of years (Willis 1685). At this point, they are recommended for those with refractory or augmented RLS (Winkelman et al. 2025b; Silber et al. 2021). In one large study of refractory RLS patients, oxycodone‐naloxone at doses of roughly 20 mg per day was superior to placebo in addressing RLS symptoms. Furthermore, a number of longitudinal observational studies demonstrate the efficacy of opioids, particularly methadone, in patients with augmented RLS, with little dose escalation over a follow‐up of 5–10 years (Winkelman et al. 2023; Walters et al. 2001; Silver et al. 2011). Side effects of these medications in RLS patients include constipation, daytime sleepiness, and sweating.

Recently, bilateral high‐frequency peroneal nerve stimulation of 30 min duration has demonstrated efficacy for medication‐refractory RLS in short trials. Side effects include minor irritation at the stimulation site (Bogan et al. 2023).

### 2.5 RLS Genetics: Insights Into New Drug Targets

The path to approval of a new drug is lengthy and expensive. Many candidates fail clinical trials and do not make it through to approval. The perspective of high investment coupled with high risk of failure likely contributes to the low interest of companies in developing new drugs specifically for RLS. Integration of genetic information can increase the chance of success. Several retrospective analyses of clinical trial results have shown that drugs whose targets had genetic evidence of being linked to a disease were about 2–3 times more likely to be approved (Minikel et al. 2024; Nelson et al. 2015; Ochoa et al. 2022).

For common diseases such as RLS, evidence linking genetic variants to the disease stems from genome‐wide association studies (GWAS). However, the corresponding association signals are usually non‐coding variants, and each identified GWAS risk locus contains several genes, which makes it challenging to identify the true causal genes. Integration of statistical fine mapping and data from functional genomics studies on gene expression and gene regulation can be used to prioritise likely candidate genes in GWAS loci (Cano‐Gamez and Trynka 2020; Reay and Cairns 2021). For RLS, GWAS have identified a total of 164 genomic risk loci to date (Schormair et al. 2024; Akçimen et al. 2024; Didriksen et al. 2020). The most recent GWAS study performed gene prioritisation in all 164 risk loci and provides a list of genes with their corresponding prioritisation score (Table S18 in (Didriksen et al. 2020)). This list can serve as a starting point for screening candidates for drug development specifically targeted to RLS. The top 228 genes of this list (ranked by their score) include 58 genes that are considered as druggable, that is encoding proteins that can or are predicted to interact with existing drugs or compounds usable as drugs (Finan et al. 2017). A subset of these genes encodes targets of approved drugs, suggesting that these could be evaluated for their suitability as additional treatment options for RLS. These include the genes encoding ferroportin (*SCL40A1*) as well as glutamate‐AMPA receptors (*GRIA1* and *GRIA4*), which are targets of effective RLS medications (see above). In‐depth assessment of all candidate genes is warranted to maximise the potential gains from these genetic analyses for developing specific drugs for RLS.

## 3 Other Sleep Related Movement Disorders

### 3.1 Restless Sleep Disorder in Children and Adults: Recent Advances in Research

Restless Sleep Disorder (RSD) is a recently defined paediatric sleep movement disorder characterised by frequent, large‐muscle movements during sleep, leading to significant sleep fragmentation and daytime impairment (Broström et al. 2023). Initially recognised in children, emerging research now suggests that similar movement patterns may be present in adults, broadening the scope of investigation (Ferri et al. 2021).

#### 3.1.1 RSD in Children

RSD primarily affects children aged 6–18 years, with an estimated prevalence of 7.7% in sleep clinic referrals (Ibrahim et al. 2023). Unlike RLS or Periodic Limb Movement Disorder (PLMD), RSD is characterised by large, non‐rhythmic body movements occurring throughout sleep (Mogavero, Congiu, Lanza, Ferini Strambi, et al. 2025). These movements contribute to sleep instability, as evidenced by changes in cyclic alternating pattern (CAP) analysis, increased arousals, and heightened sympathetic activation during sleep stages such as N3 and REM (Mogavero et al. 2024; Mogavero, Congiu, Lanza, Marelli, et al. 2025). A key pathophysiological factor in RSD is iron deficiency, with low ferritin levels frequently observed in affected children (DelRosso, Ferri, et al. 2020). Treatment primarily involves oral and intravenous iron supplementation, with intravenous iron demonstrating greater efficacy in symptom reduction and ferritin restoration (Wang et al. 2024).

RSD in children is often associated with comorbid conditions, particularly attention‐deficit/hyperactivity disorder (ADHD), parasomnias (e.g., sleepwalking, night terrors), and epilepsy (Ferri et al. 2021; DelRosso and Ferri 2019; DelRosso, Hartmann, et al. 2020). Studies indicate a bidirectional relationship between ADHD and RSD, where sleep disturbances exacerbate attentional deficits and vice versa. Additionally, children with epilepsy have a higher prevalence of RSD, with increased nocturnal arousals and disrupted sleep architecture (DelRosso and Ferri 2019).

#### 3.1.2 RSD in Adults

Recent investigations suggest that RSD may extend beyond childhood. While RLS and PLMD are well‐documented sleep movement disorders in adults, some individuals exhibit large body movements that do not conform to these conditions (DelRosso, Bruni, and Ferri 2020). A study analysing large muscle movements in adults has identified a subgroup with movement patterns resembling RSD, raising the possibility of an adult counterpart to the disorder (Ferri et al. 2021). However, diagnostic criteria and prevalence estimates in adults remain under investigation.

#### 3.1.3 Recent Research and Future Directions

Current research continues to refine the understanding of RSD. Neurophysiological studies indicate increased autonomic dysregulation, altered sleep spindles, and CAP instability in affected children (Mogavero et al. 2024). Additionally, efforts to standardise the scoring of large muscle movements have led to the development of new large muscle movement scoring criteria by the International Restless Legs Syndrome Study Group (IRLSSG) (Mogavero, Congiu, Lanza, Ferini Strambi, et al. 2025). Future research should focus on the natural history of RSD, potential biomarkers, and therapeutic advancements, including alternative pharmacological interventions.

RSD remains a distinct, clinically significant disorder requiring greater awareness and further exploration, particularly in adult populations, to enhance diagnosis and treatment strategies across all age groups.

### 3.2 Neck Myoclonus: More Than a Physiological Phenomenon

Neck myoclonus (NM) is characterised as a vertical “stripe‐shaped” movement‐induced artefact on EEG leads during polysomnography. Frauscher et al. first reported and observed it in 54.6% of a cohort of 205 patients with mixed sleep disorders during REM sleep, including insomnia, sleep‐related breathing disorders, restless leg syndrome, periodic leg movements during sleep, RBD, and narcolepsy (Frauscher et al. 2010). In a subsequent study of healthy individuals, NM was also found in 35% of 100 healthy sleepers aged 19–77 years during REM sleep, with a median index of 2/h of REM sleep, and a higher index in men compared to women (Frauscher et al. 2014).

In the whole sleep period, NM can occur both in REM and NREM sleep, but the frequency is higher in REM (Frauscher et al. 2010; Wolfensberger et al. 2019). If a characteristic “stripe‐shaped” EEG artefact is observed during video‐polysomnography, the synchronised video should be examined to determine whether visible neck myoclonus is present. NM typically appears on video as a sudden myoclonic dorsal or ventral flexion, or version of the head to one side (Frauscher et al. 2010, 2014). These events are brief, lasting less than 2 s, and may occur with or without simultaneous movements of other body parts or subsequent arousals (Frauscher et al. 2010; Wolfensberger et al. 2019). The clinical manifestation of NM varies among subjects; however, within an individual, the pattern of NM tends to be relatively consistent throughout the night. These events are generally unsustainable or nonperiodic. A case series of three subjects with periodic neck myoclonus during sleep (PNMS) was reported, with NM mainly occurring during NREM sleep, and the authors suggested that PNMS may be linked to PLMS (Pérez‐Carbonell et al. 2017). Moreover, in addition to identifying NM with obvious EEG artefacts, neck surface muscle EMG (e.g., surface EMG of bilateral sternocleidomastoid or trapezius muscles) has been shown to be useful in enhancing the detection of neck electromyographic activity, potentially improving both the sensitivity and specificity for identifying NM (Hu et al. 2021).

The mechanism of NM remains unclear. Due to the common finding of NM in V‐PSGs and its common presence in healthy sleepers, it has been historically considered as a physiological phenomenon. However, recent studies have proposed redefining neck myoclonus as ‘sleep‐related head jerk’ (SRHJ) and classifying it as a new sleep‐related movement disorder, since the event in video usually induces head jerks and the duration of NM (approximately 0.5 s) is longer than a typical cortical myoclonus (Wolfensberger et al. 2019). Furthermore, it has been suggested that SRHJ should be classified to differentiate between physiological and pathological presentations based on intensity, frequency, and comorbidities. In severe cases with a high SRHJ index (more than 30/h) during REM sleep, excessive movements might induce sleep fragmentation or sleep disturbances, and daytime sleepiness (Xu et al. 2024). Another study in patients with narcolepsy showed that excessive NM affected sleep quality and aggravated daytime sleepiness, and that the nocturnal PSG NM index was positively correlated with the number of sleep onset REM periods in the subsequent multiple sleep latency test in patients with narcolepsy type 1 (Xu et al. 2024). Therefore, the clinical significance of NM or SRHJ might need to be reconsidered. Given the high prevalence of myoclonic arousals (up to 43.7%–80%) and the non‐restorative sleep or sleep complaints that may result from excessive NM (Wolfensberger et al. 2019; Xu et al. 2024; Lopez et al. 2021; Nair et al. 2021), future studies should aim to clarify the underlying pathological mechanisms of excessive NM, the threshold for clinical relevance, and explore its potential associations with other disorders.

In general, mild cases of neck myoclonus do not need specific treatment. In some severe cases which significantly affect sleep quality, medications may be required. A previous study showed that patients treated with clonazepam did not appear to have NM (Frauscher et al. 2010); however, its efficacy in managing severe cases is not known. To date, there are no studies evaluating pharmacological treatments for NM.

### 3.3 Fragmentary Myoclonus

Fragmentary myoclonus (FM) was first described by De Lisi in 1932 and then further characterised and defined by Broughton and Tolentino in 1984 (De Lisi 1932; Broughton and Tolentino 1984). Excessive FM (EFM) is defined as very brief potentials in surface electromyography recorded during polysomnography, resulting in invisible or hardly visible twitches or jerks (Broughton and Tolentino 1984). It is an incidental finding and is listed among “Sleep Related Movement Disorders—Isolated symptoms and normal variants” in the ICSD‐3‐TR (American Academy of Sleep Medicine 2023). The definition of EFM is arbitrary, requiring documentation of ≥ 20 min of non‐rapid eye movement sleep with ≥ 5 FM potentials per minute. Evidence suggests that EFM is frequent even in healthy sleepers (prevalence of 24% in a cohort of 500 consecutive sleep laboratory patients) (Bergmann, Stefani, Ibrahim, Anselmi, et al. 2024; Bergmann, Stefani, Ibrahim, Brandauer, et al. 2024) and is present in both sleep and wakefulness (Frauscher et al. 2011). Indeed, in healthy sleepers, the median FM index was highest in REM sleep, followed by N1 and N2 sleep, and was lowest in N3 sleep (Frauscher et al. 2014). Associations were shown with increasing age, male sex, respiratory parameters, the periodic leg movements of sleep index, as well as several neurodegenerative diseases such as Machado‐Joseph disease/spinocerebellar ataxia type 3, Parkinson's disease, or REM sleep behaviour disorder (Frauscher et al. 2014, 2011; Bergmann, Stefani, Ibrahim, Anselmi, et al. 2024; Baldelli and Provini 2021). Also, EFM is more frequent in the lower extremities than in the upper extremities (Bergmann, Wanschitz, et al. 2023), corroborating the association of EFM with peripheral nerve pathology and PLMS (Bergmann, Stefani, Ibrahim, Anselmi, et al. 2024; Raccagni et al. 2016). Nerve conduction/electromyographic studies showed that 50% of a case series of 100 consecutive patients with EFM had electrophysiological anomalies such as polyneuropathy, nerve root lesions, and benign fasciculations (Raccagni et al. 2016). Furthermore, it was shown that positive airway pressure treatment might influence EFM in patients with sleep‐related breathing disorders (Bergmann, Stefani, Ibrahim, Brandauer, et al. 2024). In most cases, patients are not aware of fragmentary myoclonus and do not complain; therefore, it requires no treatment. Manual scoring of EFM is time consuming, subjective, and lacks reproducibility as needed for large‐scale clinical trials. Recently, automated quantification has become available to facilitate the scoring process (Bergmann, Högl, et al. 2023). This algorithm showed substantial agreement with a single rater in polysomnographies from 10 patients.

In summary, recent evidence suggests that FM is frequent in sleep laboratory patients (Bergmann, Stefani, Ibrahim, Anselmi, et al. 2024). It is more common in lower than upper limbs (Bergmann, Wanschitz, et al. 2023), goes along with peripheral nerve dysfunction (Raccagni et al. 2016), and can be reduced by positive airway pressure treatment (Bergmann, Stefani, Ibrahim, Brandauer, et al. 2024). Objective diagnosis can now be achieved using automatic algorithms (Bergmann, Högl, et al. 2023). If present, neurophysiological evaluation is suggested as electrophysiological abnormalities are present in 50% of cases (Raccagni et al. 2016). Future research will eventually allow clarifying if the current arbitrary cutoffs are valid, or if they will need adjustment based on data‐driven results from large‐scale multicenter studies.

### 3.4 Other Minor Sleep‐Related Motor Disorders

In recent decades, the widespread adoption of video‐polysomnographic (VPSG) techniques has significantly improved the characterisation of previously unrecognised sleep‐related movement disorders, including propriospinal myoclonus (PSM) at the wake–sleep transition and facio‐mandibular myoclonus (FMM) during sleep. However, advancements in the semiology and clinical description of these rare conditions have not always been accompanied by a deeper understanding of their underlying pathophysiological mechanisms or by substantial progress in treatment (Montagna 2004). To date, evidence‐based data on the management of these disorders remain scarce, with therapeutic approaches largely derived from single case reports or small case series.

Propriospinal myoclonus (PSM), first described by Brown et al., manifests as repetitive, arrhythmic jerky flexion or extension of the trunk (Brown et al. 1991). Neurophysiological studies suggest that PSM originates from a spinal generator at the thoracic level, with a distinctive pattern of muscle recruitment propagating up and down along the spinal cord at a slow conduction velocity (5–15 m/s). PSM at sleep onset occurs during relaxed wakefulness just before sleep onset, highlighting the wake‐to‐sleep transition as a distinct neurophysiological, neuropsychological, and clinical stage (Montagna et al. 1997). This transitional phase, often scored as stage 1 NREM sleep, should not be confused or scored together with NREM sleep according to a monochord process of deepening vigilance level (Vetrugno et al. 2001) (Antelmi and Provini 2015).

PSM is usually idiopathic, though some cases have been linked to spinal pathology, including cervical trauma, thoracic herpes zoster, syringomyelia, multiple sclerosis, and HIV infection (Antelmi and Provini 2015). In contrast, other cases have been classified as functional movement disorders due to the absence of identifiable spinal lesions and the presence of Bereitschafts potential suggestive of psychogenic origin (Latorre et al. 2025; Erro et al. 2013). High‐quality neurophysiological recordings, incorporating simultaneous EEG and EMG monitoring of affected muscles, are essential for differentiating PSM from functional myoclonus and ensuring accurate diagnosis (Antelmi and Provini 2015; Latorre et al. 2025).

Neurophysiological studies have also been instrumental in distinguishing PSM from sleep starts, a universal feature of sleep onset that occurs in healthy individuals. While frequent or exaggerated sleep starts may contribute to severe insomnia, they remain a normal physiological phenomenon rather than a pathological disorder (Calandra‐Buonaura et al. 2014).

Similarly, VPSG plays a critical role in the evaluation of sleep‐related orofacial and masticatory movements, including FMM. FMM is characterised by EMG bursts originating in the masseter and spreading to the orbicularis oris and oculi muscles, occurring exclusively during sleep (Vetrugno et al. 2002). This condition can lead to recurrent tongue‐biting episodes, a potentially alarming symptom often mistaken for nocturnal epilepsy (Zhang et al. 2020). In such cases, VPSG is indispensable for precise differential diagnosis.

Given the rarity of these disorders, larger cohort studies employing standardised VPSG protocols are warranted. However, polysomnographic assessments must remain individualised, tailored to the specific clinical presentation of each patient.

## 4 Discussion

This review summarises recent advances in RLS and other sleep‐related movement disorders, highlighting their implications for clinical practice and future research.

Insights into RLS pathophysiology emphasise the interplay between genetic risk factors, iron dysregulation, and neurotransmitter dysfunction. Advances in RLS pathophysiology continue to inform treatment strategies beyond traditional dopaminergic therapy. Furthermore, identifying LMM during sleep as an independent marker of sleep fragmentation, beyond PLMS, in patients with RLS highlights the need to comprehensively assess motor activity in these patients. As LMM do not respond to dopaminergic treatment, this suggests that there are distinct underlying mechanisms and that alternative therapies are needed to achieve a broad‐spectrum treatment of RLS. Notably, genome‐wide association studies have revealed over 160 genetic risk loci. Prioritising potential causal genes could inform clinical trials for screening candidates for the development of drugs specifically targeted at RLS. A major shift in RLS management has been driven by the problem of augmentation, whereby symptoms paradoxically worsen with dopaminergic treatment. As preventing the development of augmentation is paramount, international guidelines now favour α‐2‐δ ligands, iron supplementation, and, in refractory cases, opioids for RLS management. Neuromodulation techniques are emerging as non‐pharmacological options.

Beyond RLS, there is growing evidence to support the clinical relevance of conditions such as RSD, NM, FM, PSM, and FMM. For RSD, initially described in children, emerging adult data suggest a broader disease spectrum. NM, historically regarded as physiological, may contribute to sleep fragmentation and daytime sleepiness when excessive, warranting reconsideration of its clinical significance. Although often incidental, FM shows associations with peripheral nerve pathology and sleep‐disordered breathing. Finally, rare disorders such as PSM and FMM remain underrecognized, and video‐polysomnography is essential for accurate diagnosis and differentiation from epilepsy or functional disorders.

## 5 Conclusions

In summary, our growing understanding of the genetic, neurophysiological, and clinical aspects of sleep‐related movement disorders is enhancing diagnostic approaches and treatment strategies. Future RLS research should focus on integrating genetic risk profiling, improving phenotyping, and developing targeted therapies that address mechanisms beyond dopaminergic dysfunction. This will enable a comprehensive and personalised treatment approach to be achieved. Improving awareness and refining the diagnostic criteria could increase understanding of the clinical relevance of other sleep‐related movement disorders, thereby improving the quality of life for affected patients.

## Author Contributions

**Ambra Stefani:** conceptualization, writing – original draft, writing – review and editing, supervision. **Qi Tang:** writing – original draft, writing – review and editing. **Stefan Clemens:** writing – original draft, writing – review and editing. **Lourdes M. DelRosso:** writing – original draft, writing – review and editing. **Diego Garcia‐Borreguero:** writing – review and editing, writing – original draft. **Raffaele Ferri:** writing – original draft, writing – review and editing. **Birgit Frauscher:** writing – original draft, writing – review and editing. **Evi Holzknecht:** writing – original draft, writing – review and editing. **Federica Provini:** writing – original draft, writing – review and editing. **Barbara Schormair:** writing – original draft, writing – review and editing. **John Winkelman:** writing – original draft, writing – review and editing. **Birgit Högl:** conceptualization, writing – original draft, writing – review and editing, supervision.

## Conflicts of Interest

The authors declare no conflicts of interest.

## Data Availability

Data sharing not applicable to this article as no datasets were generated or analysed during the current study.
